# Four Weeks of 16/8 Time Restrictive Feeding in Endurance Trained Male Runners Decreases Fat Mass, without Affecting Exercise Performance

**DOI:** 10.3390/nu13092941

**Published:** 2021-08-25

**Authors:** Ashley P. Tovar, Christine E. Richardson, Nancy L. Keim, Marta D. Van Loan, Brian A. Davis, Gretchen A. Casazza

**Affiliations:** 1 Department of Nutrition, University of California, Davis, CA 95616, USA; cerrichardson@ucdavis.edu (C.E.R.); nancy.keim@usda.gov (N.L.K.); martavanloan@yahoo.com (M.D.V.L.); 2 USDA, ARS, Western Human Nutrition Research Center, Davis, CA 95616, USA; 3 Physical Medicine and Rehabilitation, University of California Davis Medical Center, Sacramento, CA 95816, USA; badmd308@gmail.com; 4 Department of Kinesiology, Sacramento State University, Sacramento, CA 95819, USA; gcasazza@csus.edu

**Keywords:** time restricted feeding, runners, sport performance, 16/8 diet, intermittent fasting

## Abstract

Background: Time restricted Feeding (TRF) is a dietary pattern utilized by endurance athletes, but there is insufficient data regarding its effects on performance and metabolism in this population. The purpose of this investigation was to examine the effects of a 16/8 TRF dietary pattern on exercise performance in trained male endurance runners. Methods: A 4-week randomized crossover intervention was used to compare an 8-h TRF to a 12-h normal diet (ND) feeding window. Exercise training and dietary intake were similar across interventions. Runners completed a dual-energy X-ray absorptiometry (DXA) scan to assess body composition, a graded treadmill running test to assess substrate utilization, and ran a 10 km time trial to assess performance. Results: There was a significant decrease in fat mass in the TRF intervention (−0.8 ± 1.3 kg with TRF (*p* = 0.05), vs. +0.1 ± 4.3 kg with ND), with no significant change in fat-free mass. Exercise carbon dioxide production (VCO_2_) and blood lactate concentration were significantly lower with the TRF intervention (*p* ≤ 0.02). No significant changes were seen in exercise respiratory exchange ratio or 10 km time trial performance (−00:20 ± 3:34 min:s TRF vs. −00:36 ± 2:57 min:s ND). Conclusion: This investigation demonstrated that adherence to a 4-week 16/8 TRF dietary intervention decreased fat mass and maintained fat-free mass, while not affecting running performance, in trained male endurance runners.

## 1. Introduction

Periodizing caloric and macronutrient intake based on training goals, duration, and intensity has been shown to optimize exercise adaptation, performance, and recovery [1]. However, athletes may deviate from this recommendation and use other nutritional strategies depending on the goals of the training block and the desired training adaptation. For example, in an effort to decrease body fat, improve fat utilization, and stimulate mitochondrial biogenesis, a lower carbohydrate diet or fasted exercise might be utilized during the off-season when exercise intensity is low [2,3]. However, longer durations of adherence to a low-carbohydrate diet may impair sport performance by limiting the body’s sources of fuel and suppressing activation of glycolytic enzymes necessary for high intensity exercise [4,5]. Time restricted feeding (TRF) has the potential to be utilized as a diet to enhance sport performance in that increasing the time spent fasting may allow for longer periods of low-carbohydrate availability to elicit these desirable training adaptations, while still providing the energy and carbohydrate needed to maintain higher intensity training during feeding periods.

A TRF dietary pattern is a form of intermittent fasting, where individuals incorporate longer than normative daily fasting periods that may extend to 10–21 h and are traditionally defined as complete caloric restriction while allowing for water intake [6]. While there are many versions of TRF, one that has gained popularity among athletes is the 16/8, which requires 16 h of fasting and 8 h of eating in a 24-h period. A TRF diet has shown favorable effects on body composition, blood pressure and insulin sensitivity in sedentary obese persons [7,8] and normal weight individuals [9], along with reducing fat mass in resistance trained athletes [10,11]. However, at present, there is little research evidence using endurance trained athletes, yet this is an important athletic group to evaluate in order to determine the impact of TRF on athletic performance.

A recent parallel designed investigation focused on the effects of 8 weeks of adherence to a 16/8 diet or isocaloric control diet, on training adaptations in resistance trained male athletes [10]. Subjects maintained their strength and fat-free mass while experiencing a significant decrease in fat mass compared to the control group (12-h feeding window). Translation of these favorable body composition changes, seen in resistance trained athletes, to endurance runners could improve exercise performance and fuel economy as a result of reducing the body mass which needs to be accelerated in this weight-bearing exercise [12]. There are potential benefits to endurance running performance as a result of extending time in a fasted state. Low energy states from fasting or depleted fuel stores after prolonged exercise may improve endurance training adaptations through increasing fat oxidation [13]. Although there is a greater reliance on carbohydrate in the form of glycogen as exercise intensity increases, adaptations to endurance training allow an athlete to better utilize fatty acids as a fuel source at the same given workload, preserving glycogen for times of necessity [14]. The ability to increase fat oxidation at a given intensity can occur from endurance training alone, but has also been demonstrated to occur in mice on a TRF diet [14,15]. Combining prolonged endurance exercise with the 16/8 TRF diet has the potential to provide synergistic effects and improve exercise performance in endurance trained athletes, as both stimuli have the ability to elicit these metabolic adaptations. Despite these hypothesized benefits that endurance runners may experience from a TRF dietary pattern, the one investigation to date that has examined the effects of a 16/8 diet on exercise performance in male runners found significant weight loss in the TRF diet group without an effect on running performance [16]. That particular investigation used a parallel design and did not control caloric intake, resulting in the TRF group consuming significantly less calories as an effect of the treatment. Investigating self-chosen dietary differences when eating windows are limited provides great value to the literature and furthers our understanding of how TRF may alter intuitive dietary intake. However, it should be noted that lowering caloric intake may have led to decreases in available energy causing physiological effects pertaining to sport performance [17]. The effects of decreased caloric intake may result in the masking of potential performance gains compared to what would be observed if caloric intake were to remain similar to normative patterns.

The substrates used during running vary greatly by intensity and to our knowledge there is no literature examining the effect of a TRF dietary pattern on substrate utilization during differing levels of running intensities [14]. Therefore, the purpose of this investigation was to determine the effects of an isocaloric 16/8 TRF diet intervention compared to a normal feeding window of 12 h, using a randomized, longitudinal crossover study design, on body composition, exercise metabolism and endurance performance in trained long-distance runners. We hypothesized that adherence to a TRF diet would increase fat oxidation and lead to subsequent decreases to whole body fat mass, and that these alterations would positively affect endurance performance.

## 2. Materials and Methods

### 2.1. Subjects

Twenty-seven healthy, endurance trained male runners between 21–36 years of age (28.7 ± 5.2 years), were recruited for this investigation by posting flyers on the UC Davis campus and from local running events and clubs. Subjects were compensated with $20.00 USD Amazon gift cards for each of the first three test visits, and $40.00 in Amazon gift cards for the final visit, totaling $100. Nine subjects did not finish the study due to personal commitments unrelated to the study protocol and three subjects were disqualified after consent. Of these, four subjects were randomized to a diet and dropped later, two were randomized to a control diet and two were randomized to the intervention of TRF. Therefore, the final sample size was fifteen, with eight subjects being randomized to TRF first. Subjects were required to have been actively training for at least 3 years, have competed in a race ≥5 km within the past 12 months, and regularly run ≥32 km·week^−1^, as this is a minimal amount of weekly mileage for most trained runners to adhere to in preparation for a 10 km race [18]. Subjects were excluded if they were taking prescription medications or dietary supplements with metabolic or cardiorespiratory effects, or were adhering to a diet defined by a >20% variation from suggested macronutrient ranges for endurance athletes as defined by the American College of Sports Medicine (3–12 g/kg/day of carbohydrate, 1.2–2.0 g/kg/day of protein, and ≥20% of caloric intake from fat) [1]. Subjects were also excluded if they had experienced any injuries in the last 3 months that prevented or limited the ability to exercise, smoked, or reported any chronic diseases affecting endocrine function, metabolism, cardiorespiratory function, or bone health. Subjects were also excluded if they presented contra-indications to exercise testing as evaluated by the study physician during their familiarization visit. Prior to participation in the investigation protocols, participants completed written informed consent approved by the institutional review board of the University of California Davis, IRB protocol number 1,223,350. The protocol was listed as identifier NCT03569852 by the national clinical trials public website.

### 2.2. Experimental Design

This cross-over intervention randomly assigned subjects to initiate the study with either a traditional 12-h feeding window (12/12) (ND) or a time restricted 8-h feeding window (16/8) (TRF). A cross-over intervention was chosen, as competitive runners have wide variations in training practices that may induce a training or de-training effect if altered. Each pattern was adhered to for 4 weeks and the participants were instructed to consume isocaloric diets of the same macronutrient composition based on their self-selected normative dietary intake patterns for each arm of the study. Prior to data collection, each subject’s baseline diet was confirmed to be within ranges proposed by the American College of Sports Medicine (ACSM) for endurance athletes as assessed by a 3-day food log to limit vast dietary variation among subjects [1]. Methods for assessment of dietary intake are listed below. During the 12/12 pattern, subjects were instructed to consume all caloric intake within the same self-selected 12-h window daily and the 16/8 pattern required subjects to consume all meals in the same 8-h period of their choosing daily. We chose not to request similar meal times for all participants as that could have disrupted their established circadian rhythm based on normative sleep/wake and feeding cycles [19]. In addition, choosing an early vs. later afternoon feeding window has not consistently demonstrated a differential effect in humans [20]. Fasting periods outside of the feeding windows only allowed for water and non-caloric beverages such as unsweetened black coffee or plain tea [21]. At the end of the first 4 weeks of the protocol, the subjects were scheduled for a washout period where they were instructed to resume their baseline dietary patterns. This period was designed to be 2 weeks, but due to the unpredicted scheduling needs of a few subjects, some had a slightly altered washout (mean 2.1 ± 0.7 weeks). After the washout period, subjects were assigned to the other intervention for another 4 weeks, for a total involvement of about 10–14 weeks. Each subject performed their exercise training in a fasted and fed state based on their baseline preferences and habits for both arms of the study, adhering to the same training protocols for both 4-week arms. Subjects completed a familiarization visit prior to engaging in the study and then visited at baseline, after 4 weeks of the first dietary intervention, and at the start and end of the second dietary intervention, for a total of 5 test days at the Western Human Nutrition Research Center in Davis, CA.

### 2.3. Familiarization Visit

The familiarization visit included obtaining consent, a resting electrocardiogram and health history, which were reviewed by the study physician to confirm the subjects’ ability to safely complete a maximal running test. After receiving medical clearance, subjects completed a VO_2_peak test followed by a 10 km treadmill time trial. The visit ended with food log training with a registered dietitian. A VO_2_peak test utilizing a graded exercise test was conducted after a 10-min self-selected warm up on a treadmill (TMX425 medical treadmill, Trackmaster, Newton, KS, USA). A metabolic cart (TrueOne 2400, ParvoMedics, Sandy, UT, USA) was calibrated prior to each test (at flow rates 50–400 L·min^−1^ and both at room air and with a standard gas mixture of 16% O_2_ and 4% CO_2_) and used to monitor gas exchange. A monitor measured heart rate (HR) continuously (5410, Polar, Woodbury, NY, USA), and rating of perceived exertion (RPE) was assessed every 2 min using a 10 point scale [22]. Subjects’ initial running speed was determined based on recent training pace to optimize the duration of the test to 12–15 min. Speed increased by 0.8 km·h^−1^ every 2-min until volitional exhaustion with a constant grade of 1% [23,24,25]. The test was considered to measure peak if at least two of the following criteria were met: a plateau in VO_2_ with increasing workload, a maximal HR > 90% of predicted (220-age), RPE >9, or respiratory exchange ratio (RER) ≥1.10 [26,27].

### 2.4. Assessment of Dietary Intake

Following the familiarization visit, subjects recorded their baseline dietary intake for 3 days. Then, during each 4-week intervention, food and beverage consumption was assessed during three days per week (including weekdays and weekends), for each of the 4-week interventions. The specific 3 days/week of food recording during each arm corresponded with three varying levels of subjective exercise intensity. One day included high intensity exercise, one of medium intensity exercise, and one of a low intensity exercise, or a rest day. Subjects used the food logging application/website MyFitnessPal (Under Armour, San Francisco, CA, USA) to log all food, beverage, and dietary supplement intake, as well as to log meal times. This application was used as a compliance tool due to its user friendly ability to increase compliance with food logging [28,29], 96% accuracy in identifying packaged foods correctly through bar code scanning features [30], and high correlation to other research tools for analyzing caloric and macronutrient intake (energy *r* = 0.85–0.93, carbohydrate *r* = 0.85–0.93, protein *r* = 0.82–0.93, fat *r* = 0.79–0.91) [31,32]. Subjects received an individual training session with a registered dietitian nutritionist to understand how to measure food quantities and accurately log their dietary intake. Subjects were encouraged to document food immediately post-consumption and to measure food volume or weight, although this was not required. Mean values for total intake as well as for each varying level of exercise intensity day were documented. Food logs were reviewed by study investigators including a registered dietitian nutritionist for accuracy. Subjects had a weekly phone check-in with investigators to discuss and edit any potential errors in dietary reporting or to make adjustments if intakes were not demonstrating consistency between interventions.

### 2.5. Assessment of Training

Wrist worn activity monitors (Polar A370, Polar, Woodbury, NY, USA) with built-in accelerometer and photoplethysmography (HR) capabilities were worn by participants to assess physical activity for each of the 4-week interventions and the first and last 3 days of the washout period. The monitor was not utilized for accuracy of training energy expenditure, HR, or distances, but utilized to confirm that training distance was held constant between both arms of the study. Subjects self-selected a personalized 4-week training routine ≥32 km·week^−1^ based on their established training methods and asked to adhere to this for each of the two 4-week arms to prevent an unwanted training effect that could influence study conclusions.

### 2.6. Experimental Protocol for Test Days

Participants arrived at the testing facility in the early morning following an overnight fast of at least 8 h. Subjects were instructed not to exercise, to follow a consistent hydration pattern, and follow a similar meal pattern (including timing of the last meal) in the 24 h prior to testing for each visit. On each of the 4 test days the following were collected in order: body mass, body composition, substrate utilization through indirect calorimetry during a running graded exercise test, and a 10 km treadmill time trial to assess performance.

### 2.7. Anthropometric Testing

Body mass was measured with a digital scale, with shoes and accessories removed (scale-Tronix, Inc., Wheaton, IL, USA) and height with a wall-mounted stadiometer (Ayrton Stadiometer, Model S100, Ayrton Corporation, Prior Lake, MN, USA). A dual whole-body x-ray absorptiometry (DXA) scan (Hologic Discovery QDR Series 84,994, Hologic, MA, USA) was used to determine body composition. The DXA scanner was calibrated prior to each use by a trained and licensed technician. All DXA scans were analyzed by a single operator to limit variation in the assessments. All subjects were placed in a standardized position by lying supine and centered on the DXA table, aligned with the long axis of the scanner. Feet were positioned with toes together with a strap restraint to maintain proper alignment of the lower body extremities. Hands were placed flat against the scanning bed. Subject alignment was matched and confirmed for subsequent tests by using the Hologic software. Subjects completed the DXA scan prior to exercise, so as to limit vast differences in hydration status.

### 2.8. Substrate Utilization Testing

Subjects performed the same 10-min standardized warmup at a speed of their choosing between 6–11 km·h^−1^ for each test day. Substrate utilization testing included a graded exercise test with increases in speed every 3 min with a 1% incline on the same treadmill utilized for VO_2_peak testing. Each of the four stages represented the running speed at which the subject achieved 60%, 70%, 80%, and 90% of their VO_2_peak, as assessed in the familiarization visit. The same speeds were used for both arms of the study. Subjects wore a mouthpiece and nose clip connected to a metabolic cart (TrueOne 2400, ParvoMedics, Sandy, UT, USA) for collection and analysis of gas exchange including ventilation rate (VE), oxygen consumption (VO_2_), carbon dioxide production (VCO_2_), and respiratory exchange ratio (RER). Percent of energy from carbohydrate and fat oxidation was extrapolated from RER utilizing methods from Frayn [33]. HR was collected continuously with a HR monitor (5410, Polar, Woodbury, NY, USA), and RPE was assessed at the end of each stage using a 10 point scale [22]. At the completion of each stage, subjects briefly straddled the treadmill for 30–60 s in order to obtain an ear-stick for blood glucose and lactate concentrations by a licensed phlebotomist. Two drops of blood in total were used for the measurement of glucose (Aviva Plus Accu-Chek, Roche, Indianapolis, IN, USA) and lactate (Lactate Plus Meter, Nova Biomedical, Waltham, MA, USA). Subjects then walked at 4.8 km·h^−1^ for 5 min, followed by 10 min of seated rest.

### 2.9. 10 km Time Trial

Following the substrate utilization test and 10 min of rest, subjects completed a 10 km running time trial on the treadmill as quickly as possible and were instructed to treat the exercise as a competitive race. Subsequent endurance testing prior to a time trial has been established to provide a reliable method of performance assessment [34]. Subjects were blinded to speed and HR, but able to manually adjust the speed. Incline was held constant at 1%. Subjects were able to choose their clothing, have a fan and listen to music of their choosing, but conditions were matched for all trials after choosing their parameters during the first trial. HR and RPE were recorded at 3.2 km, 6.4 km, and 10 km. Subjects completed a final 5-min walking cool-down after the time trial ended.

### 2.10. Statistics

Statistical analysis was completed on data derived from within group change values from preintervention to postintervention and are presented as mean differences (∆ ± standard deviation) or a mean (±standard deviation) for single intervention measurements with JMP Pro 14.2 (SAS Institute, Cary, NC, USA). Normal distribution of data was assessed with Shapiro-Wilk tests, and if necessary statistical analysis was performed on the resulting Johnson distribution data. Linear mixed modeling was utilized for statistical analysis with the fixed effects of diet and sequence to determine if a carryover effect was present. Random effects were subject by diet interaction, with a repeated residual structure. Substrate utilization data that was performed at four levels of intensity were analyzed with a three-way interaction of diet by sequence by intensity. Significance was determined with an *p* ≤ 0.05. Post-hoc analysis was performed with the Tukey’s test. Since at the time of study initiation there were no known studies examining endurance exercise performance with TRF, we used power analysis to determine a total *n* = 16, which was necessary to achieve 80% power with an α error probability of 0.05 based on primary outcomes relating to changes to body mass and fat mass [10].

## 3. Results

### 3.1. Subject Characteristics and Dietary Patterns

Subject characteristics are found in Table 1 and dietary intake can be found in Table 2. Overall exercise training and dietary intake were similar across both interventions, with the only significant difference being the timing of the feeding window (Table 1). No significant differences were found between the 4-week interventions for weekly running mileage, total caloric intake, or total macronutrient intake. However, while on the TRF intervention, there was a trend to consume less carbohydrate on higher intensity training days (*p* = 0.07).

### 3.2. Body Composition

Dietary responses to body composition are found in Table 3. A significant main effect of diet on fat mass (*p* = 0.05) was found with a greater loss of fat mass seen in response to the TRF diet pattern (−6.5%), compared to a 0.85% increase with ND. There was also a main effect of diet on body fat percent (*p* = 0.04), with a greater loss of body fat % seen in response to the TRF diet pattern (−5.9%) in comparison to a 0.62% increase with ND. No significant change was seen in fat free mass (*p* = 0.45), with either diet. There was no significant main effect of diet from the TRF diet in comparison to the ND for body mass (*p* = 0.09). A significant sequence effect (*p* = 0.05) was seen indicating that those who were randomized to start with the TRF diet pattern had greater body fat percent loss than those who were randomized to initiate the study on the ND.

### 3.3. Incremental Exercise Test

**Figure 1:**
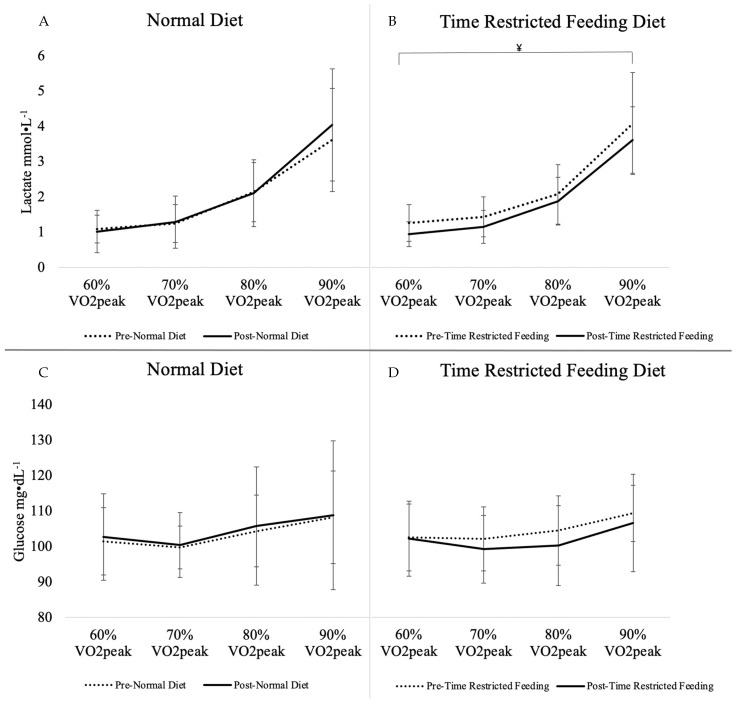
Blood Lactate and Glucose from Substrate Utilization Trial by Intensity. (**A**) Lactate response from normal diet, (**B**) Lactate response from time restricted feeding, (**C**) Glucose response from normal diet, (**D**) Glucose response from time restricted feeding. ¥, Interaction of TRF diet x intensity is significantly different from ND, *p* < 0.05. Values are means with SD, *n* = 15; VO_2_peak, peak oxygen consumption.

**Figure 2:**
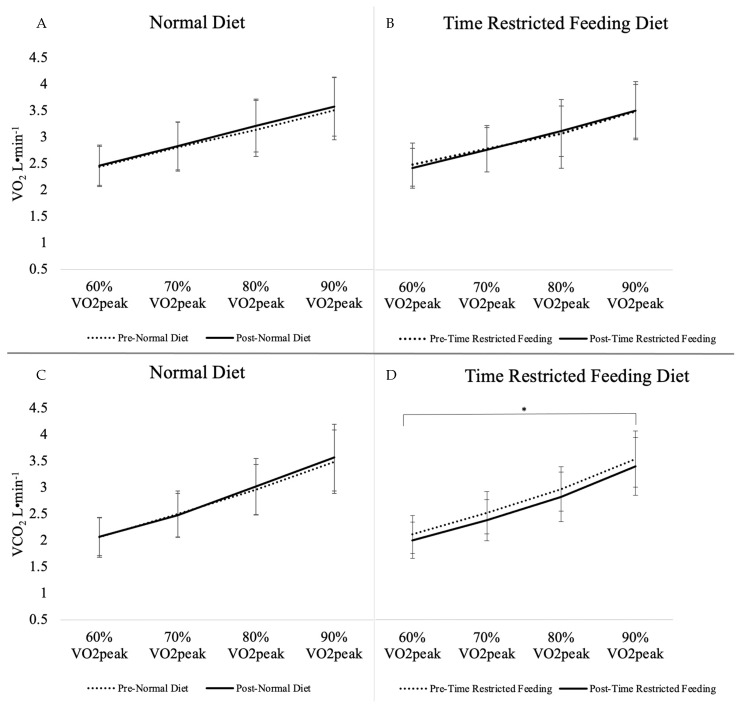
Metabolic Gases VO_2_ and VCO_2_ from Substrate Utilization Trial by Intensity. (**A**) VO_2_ response from normal diet, (**B**) VO_2_ response from time restricted feeding, (**C**) VCO_2_ response from normal diet, (**D**) VCO_2_ response from time restricted feeding. *, main effect of TRF diet is significantly different from ND, *p* < 0.05. Values are means with SD, *n* = 15; VO_2_peak, peak oxygen consumption; VO_2,_ oxygen consumption; VCO_2_, carbon dioxide consumption.

**Figure 3:**
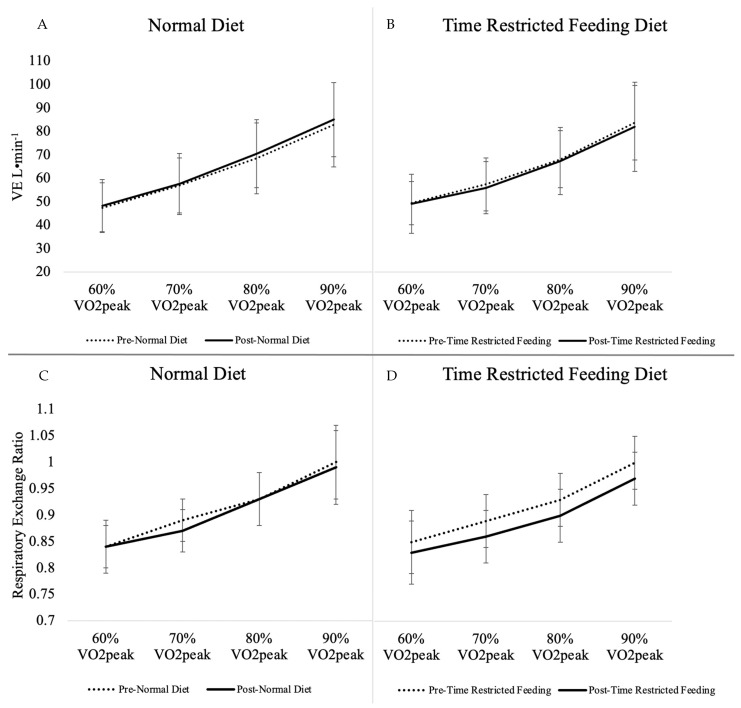
VE and Respiratory Exchange Ratio from Substrate Utilization Trial by Intensity. **A**) VE response from normal diet, (**B**) VE response from time restricted feeding, (**C**) RER response from normal diet, (**D**) RER response from time restricted feeding. Intensity values are means with SD, *n* = 15; VO_2_peak, peak oxygen consumption; VE, ventilation rate.

Gas exchange and metabolic outcomes for the main effect of diet and interaction of diet*intensity can be found in Table 4. There was a main effect of TRF diet intervention (*p* ≤ 0.01) in the change from baseline to 4 weeks on VCO_2_ during exercise_,_ which was significantly decreased when subjects were on the TRF dietary pattern (*p* ≤ 0.01). A significant sequence effect was observed for VCO_2_ (*p* = 0.03) indicating a carry-over effect as the baseline value for the second intervention had not returned to the baseline from the first intervention period. An interaction of diet by intensity was seen in blood lactate (*p* = 0.02), indicating lower lactate in response to the TRF dietary pattern compared to the ND intervention as intensity increased, specifically at the 90% VO_2_peak intensity (*p* = 0.03). Insignificant marginal decreases in VE (L·min^−1^) (*p* = 0.08), VO_2_ (L·min^−1^) (*p* = 0.09), and blood lactate (mmol·L^−1)^ (*p* = 0.07) were observed from the TRF diet in comparison to the ND (Table 3). No significant changes were seen in the main effect of diet or diet by intensity interaction for RER, % energy from carbohydrate oxidation, % energy from fat oxidation, VO_2_ (L·min^−1^), VO_2_ (mL·kg^−1^·min^−1^), VE (L·min^−1^), or blood glucose (mg·dL^−1^). Responses in cardiorespiratory outcomes, RER, blood glucose, and lactate to individual running intensities of 60% VO_2_peak, 70% VO_2_peak, 80% VO_2_peak, and 90% VO_2_peak are portrayed in Figure 1, Figure 2 and Figure 3. Respective mean ± SD speeds to these increments of peak were 9.9 ± 1.3 km·h^−1^, 11.5 ± 1.4 km·h^−1^, 13.2 ± 1.6 km·h^−1^, and 14.9 ± 1.8 km·h^−1^.

### 3.4. Performance during 10 km Time Trial

Pre- and post-time trial values are provided in Table 5. No significant differences were seen between the ND and TRF dietary pattern interventions for time to complete a treadmill 10 km distance, average HR, maximum HR, and highest RPE during the time trial.

## 4. Discussion

To our knowledge, this is the first investigation examining the effects of the 16/8 diet, in trained endurance male runners using a randomized, crossover design, while controlling for caloric and macronutrient intake. We demonstrated that adherence to a 16/8 TRF dietary pattern for 4 weeks resulted in a significant decrease in body fat percentage and fat mass while maintaining fat free mass. In addition, we observed decreased exercise expired CO_2_, and circulating blood lactate, but no change in 10 km time trial performance. Adherence to a TRF diet may have a neutral effect on running performance in that neither positive or negative effects were seen, while providing a method of decreasing fat mass during middle distance races.

One proposed benefit of TRF in regard to running is the observance that extending fasting periods may expand the contribution of fatty acids to total energy metabolism, a hallmark of endurance training [35]. However, this occurrence when fasting has mainly been observed in animal models [15,36], with mixed findings in humans [37,38]. Both fasting and endurance training initiate metabolic adaptations including increased activity of AMPK and the cyclic AMP response element-binding protein, with resulting effects to increased activity of the transcriptional regulator PPAR-α [15,39], affecting fatty acid transport and oxidation [40]. We found no significant difference in RER, or % carbohydrate oxidation, or % fat oxidation during exercise in our study. Although whole day assessments of indirect calorimetry were not included in this study, increased fat oxidation during the fasting periods of the 16/8 TRF diet is a potential hypothesis to help explain the 7% decrease in fat mass (compared to a 1% increase with a 12/12 diet), with no significant change in fat free mass. In a parallel study design with resistance trained males, Moro et al. found a similar significant 17% decrease in fat mass from a longer intervention, 8 weeks of a 16/8 TRF diet (compared to a 3% decrease with a 12/12 diet), with no difference in fat free mass change [10]. Both this investigation and our study directed participants to adhere to isocaloric diets with similar macronutrient composition in both treatments, minimizing the likelihood that body composition changes were derived from alterations to dietary intake rather than the extended fasting periods. The only other study to examine the effects of a 16/8 TRF diet in endurance athletes was an ad-libitum, 8-week parallel study design in male runners that found a significant −1.92 kg decrease in body mass compared to a control diet, but no differences were found in fat mass or fat free mass [16]. Observable differences in body composition have now been well established as a result of TRF [7,10,41]. Further research in this area should incorporate the use of stable isotope tracers to quantify flux of substrates and to expand our understanding of the underlying mechanisms of such body composition changes [42].

A potential benefit of lowering fat mass but maintaining fat free mass in a trained runner is to aid in running economy, allowing an athlete to minimize the energy demand of running at a given velocity and potentially increase performance [43]. However, despite the favorable body composition changes that were observed on the 16/8 TRF diet, this did not translate to performance benefits as no significant differences were observed in the time needed to complete the 10 km time trial, or in the rate of perceived exertion or heart rate during the time trial. In an effort to minimize equipment that may suppress running economy and effort, indirect calorimetry was not utilized during the 10 km time trial, limiting the ability to objectively quantify the effort relative to each runner’s typical aerobic capacity. The mean overall HR from the time trial was 168 ± 12 bpm. When extrapolating effort from this time trial relative to VO_2_peak effort from each subject, the time trial mean HR corresponds with the mean HR equivalent observed at 80.2 ± 0.1% of the VO_2_peak of the subjects, as determined during the familiarization visit. This effort reflects the expected oxygen uptake estimates of 75–92% VO_2_peak for distance running, with top competitors functioning ≥90% VO_2_peak [44]. This result indicates that 4 weeks of adherence to a TRF diet had neutral effects on performance for a 10 km distance event. Brady et al. did not use a time trial to assess performance, but they did not see any significant changes in running economy or VO_2_peak values during a graded maximal exercise test in their male runners after 8 weeks of a 16/8 TRF diet compared to the parallel control group [16].There was also no difference seen in the strength adaptations to an 8-week training program as a result of a 16/8 TRF diet compared to a control diet as tested separately in resistance trained male athletes and female athletes [10,11]. We know of no other study that has examined respiratory variables of metabolism during exercise up to 90% of VO_2_peak.

Liver glycogen may diminish after 12–36 h of fasting, with variation in athletic populations driven by the intensity of their training, modulating the amount of glycogen left at the beginning of such a fast [39]. The decrease of liver glycogen is associated with limitations to performance, inducing fatigue and limiting the duration an athlete can maintain a given exercise intensity, giving rise to the practice of carbohydrate loading to maximize glycogen stores prior to a racing event [45]. Extending the period of daily fasting may have led subjects to increase their total time training in a low glycogen state. However, mitochondrial biogenesis can occur through pathways that are stimulated by a low glycogen state, as AMPK has a glycogen sensing domain, limiting activity when glycogen stores are ample [46]. This indicates that the line between fatigue and performance gains remains to be established.

All subjects reported implementing a hybrid of completing their training in a fasted state with feeding shortly after their shorter runs, but completing their longer runs (>1 h) within their self-selected feeding window. Interestingly, while overall carbohydrate intake was maintained across interventions in this investigation, subjects had difficulty maintaining their normative carbohydrate intake on high intensity days (*p* = 0.07). In the investigation by Brady et al. which did not control macronutrient intake between the TRF dietary pattern and control interventions, a lower non-significant difference (*p* = 0.09) in total carbohydrate intake was also observed in these male subjects adhering to the 16/8 diet [16]. Further investigations are necessary to determine if endurance runners experience greater depletions to their glycogen stores as a result of a TRF diet expanding fasting periods, or possibly from its effects on ad-libitum carbohydrate intake, causing a resulting decrease in available glucose and, therefore, lactate during exercise. However, this is speculative and must be verified with future research.

Extreme depletions of carbohydrate availability have been shown to limit catecholamine responses, suppressing the effect of epinephrine in inducing glycogenolysis and the formation of lactate [47,48]. The hypothesis that this effect was demonstrated in this investigation may be further supported in that suppression of catecholamines as a result of a persistent lower carbohydrate availability may reduce oxidizable fuels and lead to decreases in VCO_2_, as observed in this experiment. However, circulating lactate is a function of appearance and oxidation [49]. A TRF dietary pattern has the potential to increase MCT1 transporters, allowing lactate to be shuttled to and oxidized in type I muscle fibers, which is associated with a performance benefit to heighten exercise intensity at the lactate threshold [50]. Future investigations may benefit in determining if the TRF diet’s effects on lowering blood lactate and expired VCO_2_ are related to changes in lactate transporter abundances.

The cumulative benefits of TRF outlined in this investigation demonstrate the ability of TRF to support the lowering of fat mass over time without impairing middle distance running performance. This may indicate that the best application of TRF would be for training blocks composed of mainly steady state runs at intensities similar to those utilized in a 10 km race. The indication that a TRF diet may lower lactate at higher intensities (90% VO_2peak_) suggests that performance during longer duration events requires a greater total contribution of carbohydrate as a fuel. Therefore, a ≥21.1 km race, and shorter duration events requiring a higher reliance on glycolytic type IIa muscle fibers, such as a 5 km race, may be more affected by the 16/8 diet. More research examining performance at those running distances is therefore necessary before extrapolating the results of this investigation to other distance events. In addition, a much-needed assessment in female endurance athletes who have relatively higher fat oxidation rates [51], would be warranted to determine if similar effects are seen across the sexes. It is important to note that adequate nutrition, including protein intake to support the demands associated with athletic endeavors, was achieved in the present investigation and the work by Moro et al. in male resistance trained athletes [10]. This is necessary to support the maintenance of lean mass [52], and may be a critical key in ensuring no detriments to sport performance are seen while adhering to a TRF dietary pattern. By limiting the amount of time one can spend consuming energy, a caloric deficit may occur from adherence to a TRF dietary pattern [7]. For high performing athletes, this may lead to detriments to performance, therefore the close monitoring of energy and macronutrient intake is imperative while implementing TRF into a training plan [1]. This may be of particular importance if attempting to apply this information to female endurance runners, who may be slightly more prone to experiencing symptoms of low energy availability [53]. This may lead to impairments to sport performance and long-term health, as it may be associated with alterations to hypothalamic-pituitary axis signaling if energy needs are not met [54].

The strengths of this investigation include utilizing a cross-over design to limit variation among subject characteristics and training that may lead to a confounding effect on the results. As exhibited by this investigation, a randomized crossover design has the potential flaw of inducing a sequence effect, which was seen with VCO_2_ and body fat percent, potentially reducing the impact of these results. However, no other markers in the present study were affected by a carryover effect, including fat free mass and fat mass, yet future studies examining a TRF dietary pattern should extend washout periods beyond the 2-week range used in this investigation, to avoid the potential for a sequence effect as seen with the current investigation. Due to the nature of enrolling subjects in a crossover design, intervention length was limited by the availability of trained runners (who often compete in events requiring periodized training) to maintain static training patterns during both 4-week interventions. Previous investigations on TRF have included a longer duration of intervention [10,41] (8 weeks vs. 4 weeks) and this may have blunted the potential for observable effects from a TRF diet over time, although others have seen effects on glucose and cellular markers regulating circadian rhythms and metabolism in as little as 4 days [55]. This experiment required subjects to complete a substrate utilization test prior to completing their 10 km time trial. While participation in such an exhaustive exercise bout before a race has the potential to lead to 10 km times that are not fully reflective of a real-world racing event, we felt that scheduling the testing on the same day was necessary to minimize the burden to subjects. However, this form of subsequent testing has been shown to reliably assess performance [34]. This investigation provided training for logging food, a review of dietary records by a registered dietitian for 3 days per week, and a weekly check-in to review any missing or potentially inaccurate entries by participants. However, the difficulties in assessing dietary intake accurately through various methods of assessment, including food journaling, have been well documented [56]. Therein lies the possibility that a caloric discrepancy existed in overall intake between the ND and TRF interventions that was not captured through the food logs utilized in our methods. It should also be noted that due to COVID-19 restrictions on human research, the investigation completed one subject short of the *n* = 16 sample size that was estimated to reach adequate power. The use of 15 subjects rather than 16 from the power calculation completed reduces the power to 76%. Effect size from the final data for significant findings are as follows: fat mass 0.77, body fat percent 0.75, blood lactate 0.63, and VCO_2_ 1.39.

## 5. Conclusions

The novel findings of this investigation demonstrate that adhering to a 16/8 TRF diet for 4-weeks while maintaining normative caloric and macronutrient intake had minimal effects on running performance while allowing athletes to maintain lean mass and decrease fat mass. While adherence to a TRF diet suppressed VCO_2_ and blood lactate, this did not translate to a performance effect during a 10 km time trial. These data contribute to our understanding of the appropriate application of a TRF diet as a training mechanism to induce favorable body composition changes for runners seeking to maximize their running economy without dampening performance.

## Figures and Tables

**Table 1:** Baseline Subject Characteristics from the Familiarization Visit.

Age (years)	28.7 ± 5.2
Height (cm)	177.7 ± 6.6
Weight (kg)	73.5 ± 8.6
Fat Free Mass (kg)	57.6 ± 7.6
Fat mass (kg)	12.0 ± 4.5
Body Fat (%)	16.5 ± 5.6
VO_2_peak (mL·kg·min^−1^)	55.5 ± 5.7
VO_2_peak speed (km·h^−1^)	16.3 ± 1.8
Average Running Distance (km·week^−1^)	53.0 ± 24.1
Years of Training	7.8 ± 6.0

Values are means ± SD, *n* = 15. VO_2_peak, oxygen consumption.

**Table 2:** Dietary Intake and Exercise Training Across Interventions.

	Normal Diet	TRF Diet	*p*-Value
Total caloric intake (kcal·day^−1^)	2513 ± 367	2421 ± 478	0.41
Total carbohydrate intake (g·day^−1^)	284.8 ± 79.3	269.4 ± 68.4	0.27
Total carbohydrate intake (g·kg^−1^·day^−1^)	3.9 ± 1.2	3.7 ± 1.2	0.19
Total protein intake (g·day^−1^)	112.5 ± 27.1	113.1 ± 24.4	0.42
Total protein intake (g·kg^−1^·day^−1^)	1.6 ± 0.4	1.6 ± 0.4	0.72
Total fat intake (g·day^−1^)	97.5 ± 24.5	96.8 ± 33.0	0.91
Caloric intake-HIT days (kcal·day^−1^)	2626 ± 524	2493 ± 495	0.20
Carbohydrate intake-HIT days (g·day^−1^)	307.5 ± 99.9	271.6 ± 70.0	0.07
Protein intake-HIT days (g·day^−1^)	120.1 ± 42.1	117.0 ± 27.9	0.64
Fat intake-HIT days (g·day^−1^)	97.9 ± 38.2	98.7 ± 35.9	0.94
Caloric intake-MIT days (kcal·day^−1^)	2421 ± 360	2360 ± 475	0.58
Carbohydrate intake-MIT days (g·day^−1^)	253.1 ± 71.7	271.2 ± 69.7	0.41
Protein intake-MIT days (g·day^−1^)	109.0 ± 29.0	107.6 ± 26.3	0.91
Fat intake-MIT days (g·day^−1^)	101.5 ± 27.5	92.3 ± 36.1	0.23
Caloric intake-rest days (kcal·day^−1^)	2489 ± 475	2401 ± 559	0.62
Carbohydrate intake-rest days (g·day^−1^)	293.9 ± 104.9	265.3 ± 86.3	0.18
Protein intake-rest days (g·day^−1^)	108.4 ± 29.4	115.4 ± 27.7	0.17
Fat intake-rest days (g·day^−1^)	93.1 ± 18.6	99.3 ± 34.5	0.22
Exercise (running km·week^−1^)	39.3 ± 14.2	43.0 ± 20.4	0.18
Feeding window (hours)	11.8 ± 0.6	7.6 ± 0.4	<0.01 *

Values are means ± SD, *n* = 15 *, significantly different than ND; *p* ≤ 0.05, HIT, high intensity training; MIT, medium intensity training; TRF, time restricted feeding.

**Table 3:** Body Composition.

	Pre-Normal Diet	Post-Normal Diet	Δ Normal Diet	Pre-Time Restricted Feeding	Post-Time Restricted Feeding	Δ Time Restricted Feeding	Mixed Linear Model Diet *p*-Value
Body Mass (kg)	73.0 ± 8.6	73.3 ± 8.7	+0.3 ± 1.1	73.8 ± 8.6	73.0 ± 9.0	−0.8 ± 1.9	0.09
Lean Mass (kg)	57.6 ± 7.2	58.3 ±7.8	+0.7 ± 2.4	57.7 ± 7.3	57.8 ± 7.2	+0.1 ± 1.7	0.45
Fat Mass (kg)	11.7 ± 4.8	11.8 ± 4.3	+0.1 ± 4.3	12.3 ± 4.3	11.5 ± 4.4	−0.8 ± 1.3	0.05 *
Body Fat %	16.1 ± 5.7	16.2 ± 5.3	+0.1 ± 1.3	16.8 ± 5.3	15.8 ± 5.2	−1.0 ± 1.5	0.04 *

Values are means ± SD, *n* = 15 *, significantly different than ND; *p* ≤ 0.05.

**Table 4:** Overall Cardiometabolic Variables from the Incremental Exercise Substrate Utilization Testing.

	ND Pre	ND Post	ΔND	TRF Pre	TRF Post	ΔTRF	Mixed Linear Model Diet *p*-Value	Mixed Linear Model Diet * Intensity *p*-Value
VO_2_ (L·min^−1^)	2.97 ± 0.65	3.02 ± 0.63	+0.05 ± 0.14	2.97 ± 0.62	2.97 ± 0.61	0.00 ± 0.18	0.09	0.88
VO_2_ (ml·kg·min^−1^)	39.98 ± 8.02	40.43 ± 7.92	+0.45 ± 1.50	39.95 ± 7.13	40.04 ± 7.72	+0.09 ± 1.81	0.42	0.98
VCO_2_ (L·min^−1^)	2.75 ± 0.71	2.78 ± 0.74	+0.03 ± 0.22	2.79 ± 0.68	2.66 ± 0.68	−0.13 ± 0.12	<0.01 *	0.23
VE STDP (L·min^−1^)	63.79 ± 18.76	65.37 ± 19.61	+1.58 ± 5.49	64.69 ± 17.72	63.66 ± 18.96	−1.03 ± 4.99	0.08	0.64
RPE (0-10)	3.91 ± 2.43	3.73 ± 2.25	−0.18 ± 0.91	3.80 ± 2.21	3.90 ± 2.29	+0.10 ± 1.11	0.36	0.14
HR (BPM)	159.77 ± 18.13	158. 28 ± 18.39	−1.49 ± 4.66	158.98 ± 18.48	157.74 ± 18.33	−1.24 ± 6.38	0.86	0.28
Lactate (mmol·L^−1^)	2.02 ± 1.34	2.11 ± 1.57	+0.09 ± 0.75	2.23 ± 1.45	1.91 ± 1.23	−0.32 ± 0.55	0.07	0.02 *
Glucose (mg·L^−1^)	103.50 ± 10.43	104.52 ± 15.61	+1.02 ± 13.50	104.89 ± 9.38	102.22 ± 11.56	−2.67 ± 9.87	0.19	0.58
RER	0.92 ± 0.07	0.91 ± 0.08	−0.01 ± 0.05	0.92 ± 0.08	0.89 ± 0.07	−0.03 ± 0.03	0.15	0.70
% Fat Oxidation	27.55 ± 26.36	30.30 ± 27.26	−2.75 ± 17.89	28.06 ± 25.79	37.48 ± 24.93	+9.42 ± 10.60	0.11	0.81
% Carbohydrate Oxidation	72.45 ± 26.36	69.70 ± 27.26	−2.75 ± 17.89	71.94 ± 25.79	62.52 ± 24.93	−9.42 ± 10.60	0.11	0.81

Values are means ± SD, *n* = 15 *, significantly different than ND; *p* ≤ 0.05, RER, respiratory exchange ratio, RPE, rating of perceived exhertion, VO_2_, oxygen consumption, VCO_2_, carbon dioxide consumption, VE, ventelation rate, HR, heart rate.

**Table 5:** 10 km Time Trial Parameters.

	ND Pre	ND Post	ΔND	TRF Pre	TRF Post	ΔTRF	Mixed Linear Model Diet
Time (min:sec)	50:02 ± 10:33	49:26 ± 10:04	−00:36 ± 2:57	48:42 ± 8:39	48:22 ± 9:24	−00:20 ± 3:34	0.53
Average HR (bpm)	167.8 + 11.9	169.5 ± 10.6	+1.7 ± 4.7	170.5 ± 8.5	167.6 ± 12.3	−2.9 ± 13.9	0.20
Maximal HR (bpm)	179.0 ± 13.3	181.8 ± 6.9	+2.8 ± 8.3	181.4 ± 7.5	180.8 ± 12.3	−0.6 ± 11.8	0.40
Peak RPE (0–10)	7.6 ± 2.3	7.3 ± 2.2	−0.4 ± 1.3	7.3 ± 1.9	7.3 ± 1.8	0.0 ± 1.3	0.41

Values are means ± SD, *n* = 15, RPE, rate of perceived exertion, HR, heart rate.
